# Biphasic MERS-CoV Incidence in Nomadic Dromedaries with Putative Transmission to Humans, Kenya, 2022–2023

**DOI:** 10.3201/eid3003.231488

**Published:** 2024-03

**Authors:** Brian Maina Ogoti, Victor Riitho, Johanna Wildemann, Nyamai Mutono, Julia Tesch, Jordi Rodon, Kaneemozhe Harichandran, Jackson Emanuel, Elisabeth Möncke-Buchner, Stella Kiambi, Julius Oyugi, Marianne Mureithi, Victor M. Corman, Christian Drosten, Samuel M. Thumbi, Marcel A. Müller

**Affiliations:** University of Nairobi, Nairobi, Kenya (B.M. Ogoti, V. Riitho, N. Mutono, J. Oyugi, M. Mureithi, S.M. Thumbi);; Queen Mary University of London, London, UK (V. Riitho);; Charité–Universitätsmedizin Berlin, Berlin, Germany (J. Wildemann, J. Tesch, J. Rodon, K. Harichandran, J. Emanuel, E. Möncke-Buchner, V.M. Corman, C. Drosten, M.A. Müller);; Washington State University, Pullman, Washington, USA (N. Mutono, S.M. Thumbi);; Food and Agriculture Organization, Dar es Salaam, Tanzania (S. Kiambi);; Labor Berlin–Charité Vivantes GmbH, Berlin (V.M. Corman);; German Center for Infection Research, Berlin (V.M. Corman, C. Drosten, M.A. Müller);; University of Edinburgh, Edinburgh, Scotland, UK (S.M. Thumbi)

**Keywords:** MERS-CoV, Middle East respiratory syndrome coronavirus, viruses, zoonoses, dromedary camel, reservoir, MERS, coronavirus, dynamics, incidence, Kenya

## Abstract

Middle East respiratory syndrome coronavirus (MERS-CoV) is endemic in dromedaries in Africa, but camel-to-human transmission is limited. Sustained 12-month sampling of dromedaries in a Kenya abattoir hub showed biphasic MERS-CoV incidence; peak detections occurred in October 2022 and February 2023. Dromedary-exposed abattoir workers (7/48) had serologic signs of previous MERS-CoV exposure.

Middle East respiratory syndrome coronavirus (MERS-CoV) is endemic in dromedary camels from the Arabian Peninsula and Africa; seroprevalence is >75% (*1*–*3*). Zoonotic transmission to humans has occurred sporadically, mainly on the Arabian Peninsula; >2,400 MERS cases and >800 deaths have occurred (*4*). Despite Kenya being a major camel-breeding country, only 3 potentially autochthonous camel-exposed humans with subclinical MERS-CoV infections were identified in 2019 (*5*). The apparent regional epidemiologic differences might be linked to factors such as limited diagnostics, local risk factors (e.g., human comorbidities, camel herding practices, seasonality), or MERS-CoV strain–specific features (*6*).

In farmed dromedary camels, MERS-CoV outbreaks were associated with annually synchronized camel parturition (*7*). In particular, camel calves tested MERS-CoV RNA–positive upon the loss of maternal antibodies 4–6 months after birth. Because of seasonality and changing food availability, most camels in Africa are nomadic and have variable population density. High population density is correlated with MERS-CoV seropositivity in camels in Kenya (*1*), but detailed insights into MERS-CoV circulation are missing.

Field studies on nomadic camels are hampered by limited infrastructure in remote and resource-restricted regions (*8*). However, nomadic camels are regularly transported to abattoir hubs, enabling sustained daily testing. We performed a continuous 12-month study at an abattoir hub in northern Kenya to investigate MERS-CoV incidence in nomadic camels and explore potential transmission to slaughterhouse workers.

## The Study

**Figure 1:**
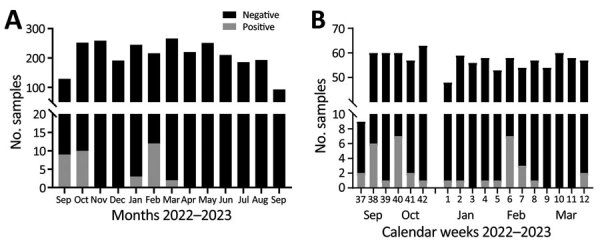
Biphasic Middle East respiratory syndrome coronavirus (MERS-CoV) incidence in dromedaries sampled in an abattoir hub, northern Kenya, 2022–2023. A) MERS-CoV RNA detection rates in nasal swab specimens from dromedary camels tested by MERS-CoV upE quantitative reverse transcription PCR. Continuous 12-month sampling (4–5 days per week) took place in Isiolo abattoir from mid-September 2022 to mid-September 2023. Sampling was suspended for 1 week in December 2022 and 1 week in July 2023. B) Detailed weekly overview of MERS-CoV RNA detections, peaking in October 2022 and February 2023.

Our sampling site was an abattoir hub in Isiolo, northern Kenya, where camels from Marsabit, Samburu, and Isiolo counties are slaughtered (Appendix Figure 1). During September 2022–September 2023, we took samples from 10–15 dromedary camels 4–5 days per week (Appendix). The camels (n = 2,711) were originally from 12 different administrative wards, mainly from Laisamis in Marsabit County (n = 1,841, 67.9%) and Burat in Isiolo County (n = 578, 21.3%) (Table; Appendix Figure 1). MERS-CoV RNA was detected in 36/2,711 (1.3%) (Table; Figure 1) camels using quantitative reverse transcription PCR, which amplifies the upstream of the envelope E gene, and confirmed by open reading frame (ORF) 1ab quantitative reverse transcription PCR or sequencing (Appendix). The cumulative RNA positivity rate was higher in September–October 2022 at 19/381 (5.0%) compared with 17/727 (2.3%) in January–March 2023 (Figure 1). Incidence was biphasic, showing detection peaks in the first weeks of October 2022 (7/60, 11.7%) and February 2023 (7/58, 12.1%) (Figure 1, panel B). For 9/36 MERS-CoV–positive samples, we obtained ORF1ab sequences and performed phylogenetic analysis. The 9 ORF1ab sequences were highly similar (>99.93% nucleotide identity) and had 99.75%–99.78% nucleotide identity with the closest MERS-CoV relative identified in Akaki, Ethiopia, in 2019 (*9*). Phylogenetic analysis showed that the 9 sequences clustered as a monophyletic group within clade C2.2, which encompasses East Africa strains initially detected in Kenya in 2018 (*10*) (Appendix Figure 2). Those sequences represent 3 putative MERS-CoV outbreaks occurring contemporarily in camels in Kenya (Appendix Table 1).

**Table:** Overview of camel samples and MERS-CoV RNA positivity in study of MERS-CoV incidence in nomadic dromedaries with putative transmission to humans, Kenya, 2022–2023*

Region	MERS-CoV RNA–positive samples/total samples (%)	Town of origin	MERS-CoV RNA–positive samples/total samples (%)	
			Female camels	Male camels
Isiolo	15/859 (1.75)	Burat	1/252 (0.4)	10/326 (3.1)
		Bulla Pesa	0/2	0/0
		Kinna	0/13	1/19 (5.3)
		Oldo/Nyiro	0/2	2/23 (8.7)
		Garbatulla	0/1	0/1
		Ngare Mara	0/106	0/114
Marsabit	26/1,846 (1.41)	Laisamis	1/250 (0.4)	21/1,591 (1.3)
		Marsabit Central	0/0	0/1
		Sololo	0/1	0/0
		North Horr	0/0	0/2
		not defined	0/0	0/1
Samburu	0/6 (0)	Wamba East	0/0	0/6
Total	36/2,711 (1.3)		2/627 (0.3)	34/2,084 (1.6)

*MERS-CoV, Middle East respiratory syndrome coronavirus.

To test whether biphasic MERS-CoV RNA–positivity is accompanied by increased MERS-CoV IgG levels, we tested randomized camel serum samples (n = 369/2,711) by MERS-CoV S1 ELISA (Appendix). MERS-CoV IgG levels showed a median optical density ratio (ODR) of 2.14 (95% CI 0.59–3.48) and a seroprevalence of 80.76% (298/369) (Appendix Figure 3, panel A). Lowest IgG levels were identified in June (median ODR 1.28, 95% CI 0.20–3.31), whereas the highest levels were seen in March (median ODR 2.72, 95% CI 1.67–3.76). MERS-CoV IgG levels were negatively associated with RNA-positivity (Odds ratio [OR] 0.20, 95% CI 0.09–0.44; p<0.0001) (Appendix Figure 3, panel B). RNA-positivity was negatively associated with the season (dry vs. wet, OR 0.14, 95% CI 0.06–0.30; p<0.0001). Male camels were more likely to be RNA positive (OR 3.94, 95% CI 0.86–29.2; p = 0.11) and less likely to be seropositive (OR 0.27, 95% CI 0.08–0.77; p = 0.021) than were female camels. Older animals (>3 years of age) were more likely to be seropositive (86%) than were animals ≤3 years of age (72%), but this difference was not statistically significant.

**Figure 2:**
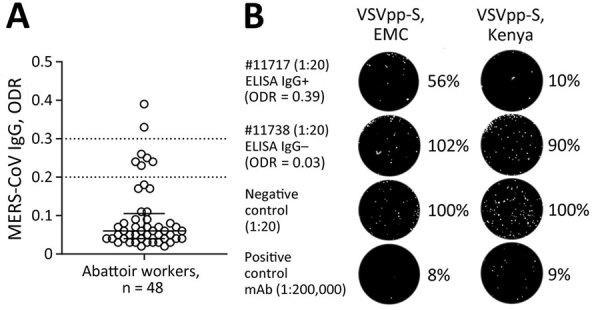
MERS-CoV immune responses in camel-exposed abattoir workers in Isiolo, Kenya. A) Results of commercial MERS-CoV S1-protein ELISA to detect IgG responses in 48 serum samples (diluted 1:100) from Isiolo abattoir workers. Samples with ODR >0.2 were considered ELISA-reactive, suggesting that 7/48 persons had MERS-CoV–reactive IgGs. Of note, all persons tested negative by MERS-CoV quantitative reverse transcription PCR. B) GFP-VSVpp-MERS-CoV S protein-based neutralization test (VSVpp-NT). VSVpp-S (EMC) and VSVpp-S (Kenya) contained human codon-optimized Spikes from prototypic MERS-CoV EMC/2012 clade A and Kenya clade C2.2 (#L00009980). All 7 ELISA-reactive human serum samples were mixed with 200 foci-forming units VSVpp in final serum dilutions 1:20–1:160. Out of the 7 ELISA-reactive persons, 1 showed a VSVpp-NT 50% foci-forming units reduction titer of 1:20 (EMC) and 1:40 (Kenya). The picture shows an example of the 1:20 dilution of an ELISA-reactive (#11717) and ELISA-nonreactive (#11738) abattoir worker. Negative control = ELISA-negative human serum (1:20) was used as reference and set to 100%. Positive control = monoclonal anti-MERS-CoV Spike receptor-binding domain binding antibody (mAb 7.7G6) previously shown to neutralize MERS-CoV at the tested dilution (1:2 × 10^5^). For better graphical visibility, all pictures were enhanced in contrast and brightness identically. mAb, monoclonal antibody; MERS-CoV, Middle East respiratory syndrome coronavirus; ODR, optical density ratio; VSVpp, vesicular stomatitis virus pseudoparticles.

Seroepidemiologic studies have suggested that abattoir workers in contact with dromedaries are at increased risk for MERS-CoV exposure (*11*). Seroconversion of subclinical MERS cases might be missed when diagnostically implemented ELISA cutoffs of commercial kits (e.g., ODR = 1.1 for IgG positives) are applied (*11*,*12*). We identified MERS-CoV S1 IgG reactivity (ODR >0.2) in 7/48 (14.6%) of Isiolo abattoir workers (Figure 2, panel A). We excluded SARS-CoV-2 infection– or vaccine–induced antibody cross-reactivity with MERS-CoV S1 by comparison of ELISA ODRs of MERS-CoV S1–based with SARS-CoV-2 S1–based ELISA (Appendix Table 2, Figure 4). A control cohort (n = 12) with no history of camel exposure showed no MERS-CoV S1 IgG reactivity (0/12; 0%) despite high SARS-CoV-2 S1 IgG levels (11/12; 92%) (Appendix Table 2).

Neutralization tests (NT) based on GFP-encoding vesicular stomatitis virus pseudoparticles (VSVpp) carrying the MERS-CoV S protein from clade A EMC/2012 or clade C2.2 (Kenya) showed that 1/7 serum samples (1:20 dilution) had a VSVpp-NT 50% reduction of foci-forming units for EMC/2012 and a 90% reduction for Kenya VSVpp-S (Figure 2, panel B). A MERS-CoV EMC/2012-based plaque-reduction neutralization test (PRNT) showed a 50% PRNT at the 1:20 dilution, fulfilling the World Health Organization criteria for a confirmed MERS-CoV seroconversion. None of 6 selected MERS-CoV S1 ELISA*-*negative abattoir samples showed neutralizing capacity when tested by VSVpp-NT and PRNT (Appendix Table 2).

## Conclusions

Our sustained sampling of dromedary camels showed a biphasic MERS-CoV incidence in northern Kenya not observed in previous studies (*1*,*10*,*13*). One explanation might be the short time of virus excretion in MERS-CoV–infected dromedaries (*14*), making viral RNA detection difficult without daily surveillance. Phylogenetic analysis suggests that we identified >3 MERS-CoV clusters over 3 different weeks in dromedaries originating from different wards. The first potential factor likely influencing the outbreaks is increased animal-to-animal interactions, because camels from different herds are transported to Isiolo and kept in holding pens together before slaughter, which could enhance MERS-CoV outbreaks. Second, increased interactions between immunologically naive and infected animals during transport and in holding pens increases the probability of transmitting MERS-CoV. That hypothesis is supported by the high percentage of IgG–negative adult camels (19.24%, ODR<0.3) (*1*,*7*). Although identifying the exact MERS-CoV transmission scenario between camels is logistically difficult, rapid point-of-care tests might help trace infections even in resource-limited conditions. 

The overall biphasic MERS-CoV incidence might be linked to seasonal factors, such as the biannual alternating wet and dry seasons in northern Kenya. During dry seasons, herds congregate using limited forage, then migrate back to the point of origin in wet seasons. Because calves are mainly born during the 2 wet seasons, the loss of protection by maternal antibodies coincides with the dry seasons. Of note, the 2 dry seasons during July–October 2022 and January–February 2023 matched the peaks of MERS-CoV RNA–positivity in October 2022 and February 2023. The combination of immunologically naive, possibly infected camel calves and the dry season–specific increased population density and probability of contact at limited waterholes might encourage MERS-CoV infections and transmissions among camels.

We identified 7/48 abattoir workers with putative MERS-CoV exposure or past subclinical infection by implementing ELISA ODR cutoffs previously shown to be suitable for seroepidemiologic studies outside clinical settings. In 1/7 cases, we confirmed MERS-CoV neutralizing antibodies by VSVpp-based NT and PRNT. None of the abattoir workers experienced severe symptoms in recent years, supporting the hypothesis that clade C strains might have limited pathogenicity and transmissibility (*15*). Identifying defined factors that drive MERS-CoV outbreaks will assist in predictive epidemiology, risk assessment, and timely precautionary interventions for public and occupational health.

Appendix Additional information about biphasic MERS-CoV incidence in nomadic dromedaries with putative transmission to humans, Kenya, 2022–2023
