# Supplementary material for: Biphasic MERS-CoV Incidence in Nomadic Dromedaries with Putative Transmission to Humans, Kenya, 2022–2023
Source: Emerg Infect Dis. 2024 Mar;30(3):581–5. doi: 10.3201/eid3003.231488 (PMC10902546; doi:10.3201/eid3003.231488)
Supplement: Appendix — Additional information about biphasic MERS-CoV incidence in nomadic dromedaries with putative transmission to humans, Kenya, 2022–2023 [file 23-1488-Techapp-s1.pdf]

Article DOI: https://doi.org/10.3201/eid3003.231488

EID cannot ensure accessibility for supplementary materials supplied by authors. Readers who have difficulty accessing supplementary content should contact the authors for assistance.

Biphasic MERS-CoV Incidence in Nomadic Dromedaries with Putative Transmission to Humans, Kenya, 2022–2023

Appendix

Material and Methods

Four-to-five days per week, 10–15 dromedary camels were sampled post-mortem from mid-September 2022 to mid-September 2023 (total n = 2,711). The majority of sampled camels were male (77%, 2,084/2,711) with a median of 3.5 years (range 2–8 years). The median age of females was 6 years (range 2–9 years). The post-mortem swabbing was performed on the caudal turbinate of the nose using Copan FLOQSwabs (Mast Diagnostica GmbH, Reinfeld, Germany), after making a transverse incision above the nostrils to avoid contaminations from the lower part of the nose. For serologic analyses, blood samples were obtained by jugular vein puncture.

A cross-sectional sampling of camel workers (n = 48) at the Isiolo abattoir was conducted in February 2023. Oropharyngeal sampling of humans was also done using Copan FLOQSwabs. For serologic testing, 5 mL blood were drawn in VACUETTE® gold cap tubes (Greiner Bio-One GmbH, Kremsmünster, Austria). In addition, a control cohort (n = 12) with no known camel contact was recruited at the University of Nairobi. Ethical approval for the collection of camel and human samples was granted by the Kenyatta National Hospital Ethics and Research Committee (protocol number P534/08/2020), and the Kenya National Commission of Science and Technology (NACOSTI) no. P/22/21987.

RNA extraction from camel and human samples was done using a MagnaPure 96-well plate nucleic acid extraction kit (Roche, Penzberg, Germany). MERS-CoV RNA was detected

and quantified by MERS-CoV upE RT-qPCR assay, as previously described (*1*). All samples were additionally confirmed by the MERS-CoV ORF1a RT-qPCR assay or sequencing (*2*). For 9 samples with MERS-CoV RNA concentrations >1 × 10^6 genome copies/mL, MERS-CoV partial genomes were generated by Illumina next-generation sequencing (NGS). Sequence assembly was done by mapping the reads to the reference MERS-CoV EMC/2012 strain (GenBank accession No. NC_019843.3) using Geneious (version 2023.2.1), Multiple Alignment using Fast Fourier Transform (MAFFT) for sequence alignment, and a Tamura-Nei, neighbor-joining model for phylogenetic inference. All 9 Orf1ab sequences were uploaded to NCBI (GenBank no. OR742168–75).

MERS-CoV-specific IgGs from dromedary and human sera were detected by a commercial MERS-CoV S1 enzyme-linked immunosorbent assay (ELISA, EUROIMMUN AG, Lübeck, Germany), as described previously (*3–6*). Serum samples were applied at a 1:100 dilution previously shown to be optimal for screening purposes (*4*). The assay-specific cutoff ranges (OD ratio = 0.2–0.3 for humans, OD ratio = 0.3 for camels) and the MERS-CoV seropositivity rating (stage 1 = ELISA-reactive; stage 2 = ELISA-reactive + virus neutralization test ≥1:20) were chosen based on our previous seroepidemiological surveys (*4*,*7*). SARS-CoV-2-specific IgGs from human sera (abattoir workers and control cohort) were detected by a commercial SARS-CoV-2 S1 ELISA (EUROIMMUN AG, Lübeck, Germany) according to manufacturer´s instruction using a 1:100 dilution and an OD ratio = 1.1 as cutoff.

For camel sample randomization, 25 camel samples from each month were selected to be representative of the complete sample set after stratification using the *strata* function in R. All MERS-CoV RNA-positive samples were not included in the randomization but were also tested by ELISA.

Human codon-optimized MERS-CoV Spike sequences of clade A EMC/2012 and clade C2.2 Kenya No. L00009980, lacking the last C-terminal 19 aa that contain an ER retention signal (*8*), were cloned into pCG1 vector using In-Fusion® Snap Assembly (TAKARA, Japan). Spike-carrying Vesicular Stomatitis Virus-pseudotyped particles (VSVpp) with a GFP reporter were generated in HEK293T cells, as previously described (*9*). Diluted sera (1:20–1:160) or the positive control mAb 7.7G6 (1:20,000–1:400,000) (*8*), respectively, were tested in triplicates and mixed with 200 foci-forming units (FFUs) of VSVpp in 96 well plate, and incubated at 37°C for

30 minutes. Following incubation, Vero B4 suspension cells (2.5×10^4 cells/well) were added to the plate and incubated at 37°C for 16 hours. Following the final incubation, culture medium was aspirated, and the cells were fixed with ROTI®Histofix (Carl Roth) for 15 minutes succeeded by 5 minutes of staining with DAPI (0.3 µg/mL, Merck) for cell confluency control. The resulting FFUs were imaged using the Autoimmun Diagnostika V Spot Spectrum, with an exposure time of 1 sec for GFP and 0.2 sec for DAPI at high resolution, and FFUs with intensity of ≥80 were counted. The same instrumental settings were used for the automatically processed and documented 96-well plates.

MERS-CoV plaque reduction neutralization test (PRNT) was performed following a previously described protocol (*10*), but using 500 PFU/mL of a recombinant MERS-CoV EMC/2012 strain stock (*11*) in a 24-well plates seeded with 3.5×10^5 cells/well 1 day before use. Serum samples were first inactivated at 56°C for 30 minutes and dilutions 1:10–1:40 were tested in duplicates. Serum-virus 1:1 dilution mixtures were incubated for 1 hour at 37°C before being applied onto VeroB4 cell monolayers. As a positive control, the same mAb against MERS-CoV Spike (mAb 7.7G6) was included in a dilution series of 1:100,000–1:400,000 (*12*). Assayed supernatants were discarded after 1 h, DMEM (Lonza, Switzerland) supplemented with 1.2% Avicel (Sigma-Aldrich, USA) was added and the cultures were incubated for 4 days at 37°C and 5% CO_2. Cells were fixed with 6% paraformaldehyde and stained with 1% crystal violet (Carl Roth, Germany). The PRNT titer was calculated based on a 50% (PRNT50) reduction in infected cells counts.

Graphing was done using GraphPad prism version 9.5.1, while statistical analysis was done on GraphPad and R software (R Studio Version 2022.12.0).

Appendix Table 1. Overview and details of MERS-CoV RNA-positive camels*

Camel ID	Sampling date	County of origin	Place of origin	Sex	MERS-CoV RNA concentration [copies/mL] upE assay	OD ratio IgG ELISA	Proposed subclusters according to phylogeny
L00009821	16.09.2022	Marsabit	Laisamis	M	1.63×10^{8}	NS	I
L00009820	16.09.2022	Marsabit	Laisamis	M	6.91×10^{4}	NS	NA
L00009375	19.09.2022	Isiolo	Burat	M	6.41×10^{3}	4.24	NA
L00009402	20.09.2022	Isiolo	Burat	M	1.02×10^{4}	3.91	NA
L00009403	20.09.2022	Isiolo	Burat	F	1.87×10^{6}	0.3	I
L00009560	21.09.2022	Isiolo	Burat	M	8.5×10^{2}	NS	NA
L00009558	21.09.2022	Marsabit	Laisamis	M	1.3×10^{7}	3.11	I
L00009662	23.09.2022	Isiolo	Oldo/Nyiro	M	4.34×10^{5}	0.06	NA
L00009804	29.09.2022	Isiolo	Oldo/Nyiro	M	2.29×10^{3}	0.06	NA
L00009954	03.10.2022	Marsabit	Laisamis	M	3.15×10^{6}	0.18	II
L00009966	04.10.2022	Isiolo	Burat	M	5.88×10^{8}	NS	II
L00009967	04.10.2022	Isiolo	Burat	M	1.82×10^{3}	0.21	NA
L00009989	06.10.2022	Isiolo	Burat	M	6.08×10^{2}	0.04	NA
L00009980	06.10.2022	Marsabit	Laisamis	M	7.4×10^{7}	0.06	I
L00009890	07.10.2022	Marsabit	Laisamis	M	1.67×10^{3}	3.84	NA
L00009896	07.10.2022	Marsabit	Laisamis	M	6.1×10^{8}	0.29	II
L00009913	11.10.2022	Isiolo	Burat	M	2.32×10^{5}	0.19	NA
L00009817	12.10.2022	Isiolo	Kinna	M	4.89×10^{5}	0.14	NA
H08150251	20.10.2022	Marsabit	Laisamis	M	5.18×10^{3}	0.31	NA
L00011101	05.01.2023	Marsabit	Laisamis	M	3.77×10^{7}	0.21	NA
L00011081	10.01.2023	Marsabit	Laisamis	M	2.95×10^{4}	0.15	NA
L00011418	24.01.2023	Marsabit	Laisamis	F	4.95×10^{6}	2.07	III
L00011505	03.02.2023	Marsabit	Laisamis	M	1.26×10^{6}	0.22	III
L00011523	06.02.2023	Isiolo	Burat	M	1.49×10^{3}	2.46	NA
L00011520	06.02.2023	Marsabit	Laisamis	M	6.22×10^{3}	0.7	NA
L00011530	07.02.2023	Marsabit	Laisamis	M	3.76×10^{4}	0.29	NA
L00011542	08.02.2023	Marsabit	Laisamis	M	4.29×10^{4}	3.81	NA
L00011543	08.02.2023	Marsabit	Laisamis	M	7.85×10^{3}	1.9	NA
L00011549	09.02.2023	Marsabit	Laisamis	M	1.36×10^{5}	0.17	NA
L00011551	09.02.2023	Marsabit	Laisamis	M	1.49×10^{3}	2.23	NA
L00011576	13.02.2023	Marsabit	Laisamis	M	3.64×10^{3}	0.67	NA
L00011595	14.02.2023	Isiolo	Burat	M	3.49×10^{4}	0.15	NA
L00011622	17.02.2023	Isiolo	Burat	M	3.26×10^{5}	0.28	NA
L00011675	23.02.2023	Marsabit	Laisamis	M	6.78×10^{4}	2.29	NA
L00011894	21.03.2023	Marsabit	Laisamis	M	2.78×10^{3}	2.51	NA
L00011930	24.03.2023	Marsabit	Laisamis	M	1.49×10^{3}	2.54	NA

*NS, no serum sample available; NA, not applicable.

Appendix Table 2. Serologic assessment of human abattoir workers and a control cohort*

Cohort	Sample ID	MERS-CoV (ELISA OD ratio)	SARS-CoV-2 (ELISA OD ratio)	Reciprocal VSVpp-NT titer 50% (EMC/Kenya Spike)	Reciprocal VSVpp-NT titer 90% (EMC/Kenya Spike)	Reciprocal PRNT50/90 based on MERS-CoV EMC	Rating for MERS-CoV seropositivity
Abattoir	11706	0.23	4.48	<20/<20	<20/<20	<10/<10	stage 1 pos.
Abattoir	11717	0.39	6.83	20/40	<20/20	20/10	stage 2 pos.
Abattoir	11722	0.33	0.62	<20/<20	<20/<20	<10/<10	stage 1 pos.
Abattoir	11727	0.25	2.63	<20/<20	<20/<20	<10/<10	stage 1 pos.
Abattoir	11732	0.24	1.28	<20/<20	<20/<20	<10/<10	stage 1 pos.
Abattoir	11741	0.26	5.13	<20/<20	<20/<20	<10/<10	stage 1 pos.
Abattoir	11746	0.24	3.02	<20/<20	<20/<20	<10/<10	stage 1 pos.
Abattoir	11712	0.03	7.61	<20/<20	<20/<20	<10/<10	negative
Abattoir	11738	0.03	4.48	<20/<20	<20/<20	<10/<10	negative
Abattoir	11753	0.03	1.89	<20/<20	<20/<20	<10/<10	negative
Abattoir	11728	0.05	6.34	<20/<20	<20/<20	<10/<10	negative
Abattoir	11734	0.08	5.30	<20/<20	<20/<20	<10/<10	negative
Abattoir	11755	0.11	2.61	<20/<20	<20/<20	<10/<10	negative
Control	83319	0.09	3.12	n.d.	n.d.	n.d.	negative
Control	83320	0.09	7.41	n.d.	n.d.	n.d.	negative
Control	83321	0.15	6.44	n.d.	n.d.	n.d.	negative
Control	83322	0.13	0.47	n.d.	n.d.	n.d.	negative
Control	83323	0.08	5.10	n.d.	n.d.	n.d.	negative

Cohort	Sample ID	MERS-CoV (ELISA OD ratio)	SARS-CoV-2 (ELISA OD ratio)	Reciprocal VSVpp-NT titer 50% (EMC/Kenya Spike)	Reciprocal VSVpp-NT titer 90% (EMC/Kenya Spike)	Reciprocal PRNT50/90 based on MERS-CoV EMC	Rating for MERS-CoV seropositivity
Control	83324	0.09	8.91	n.d.	n.d.	n.d.	negative
Control	83325	0.08	6.01	n.d.	n.d.	n.d.	negative
Control	83326	0.09	7.95	n.d.	n.d.	n.d.	negative
Control	83327	0.10	7.51	n.d.	n.d.	n.d.	negative
Control	83328	0.12	2.53	n.d.	n.d.	n.d.	negative
Control	83329	0.09	6.95	n.d.	n.d.	n.d.	negative
Control	83330	0.17	8.87	n.d.	n.d.	n.d.	negative

*Stage 1 pos., ELISA-reactive; stage 2 pos., ELISA-reactive and virus neutralization test–positive; n.d. = not done.

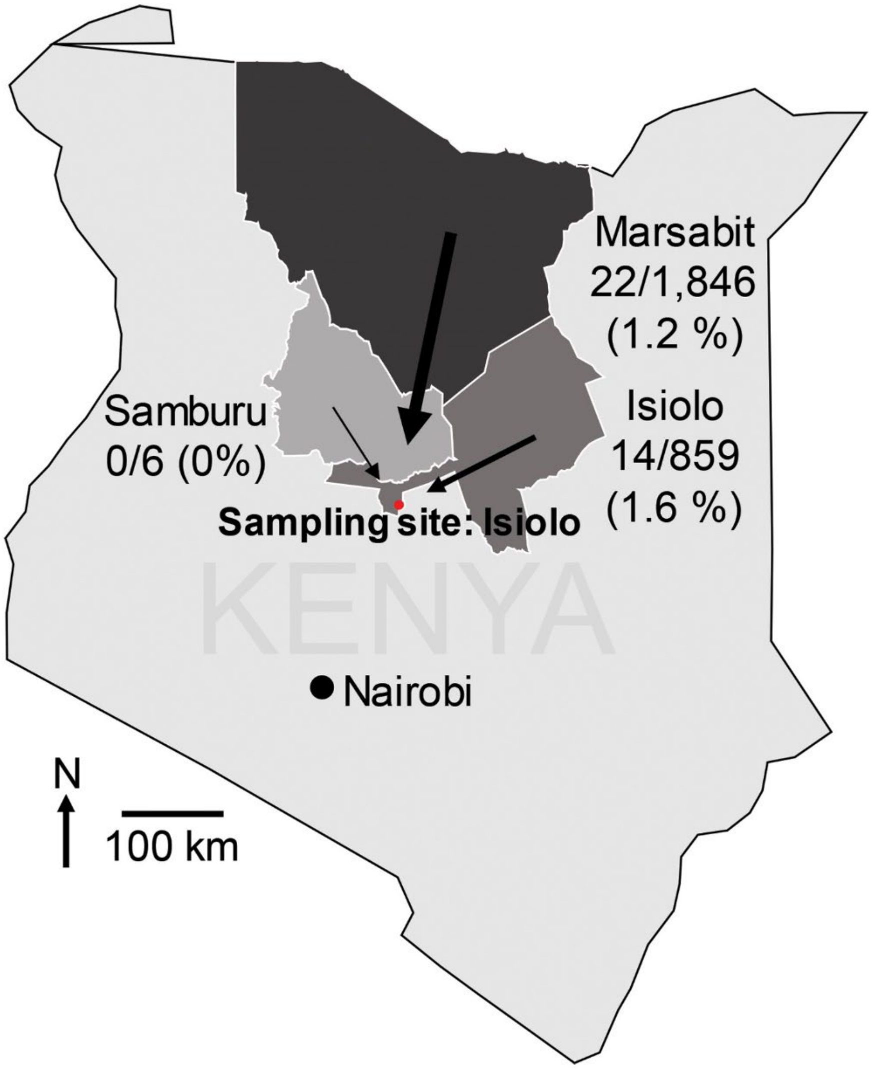

Appendix Figure 1. Origin of all MERS-CoV RNA-positive dromedaries. All dromedary samples (n = 2,711) in this study were sampled in an abattoir hub in Isiolo, where dromedaries from the sub-national regions of Marsabit, Isiolo, and Samburu are slaughtered. The number of total samples, MERS-CoV RNA-positive samples, and percentage positivity are shown for each region. Arrows indicate the direction of transportation from the regions to the Isiolo abattoir hub.

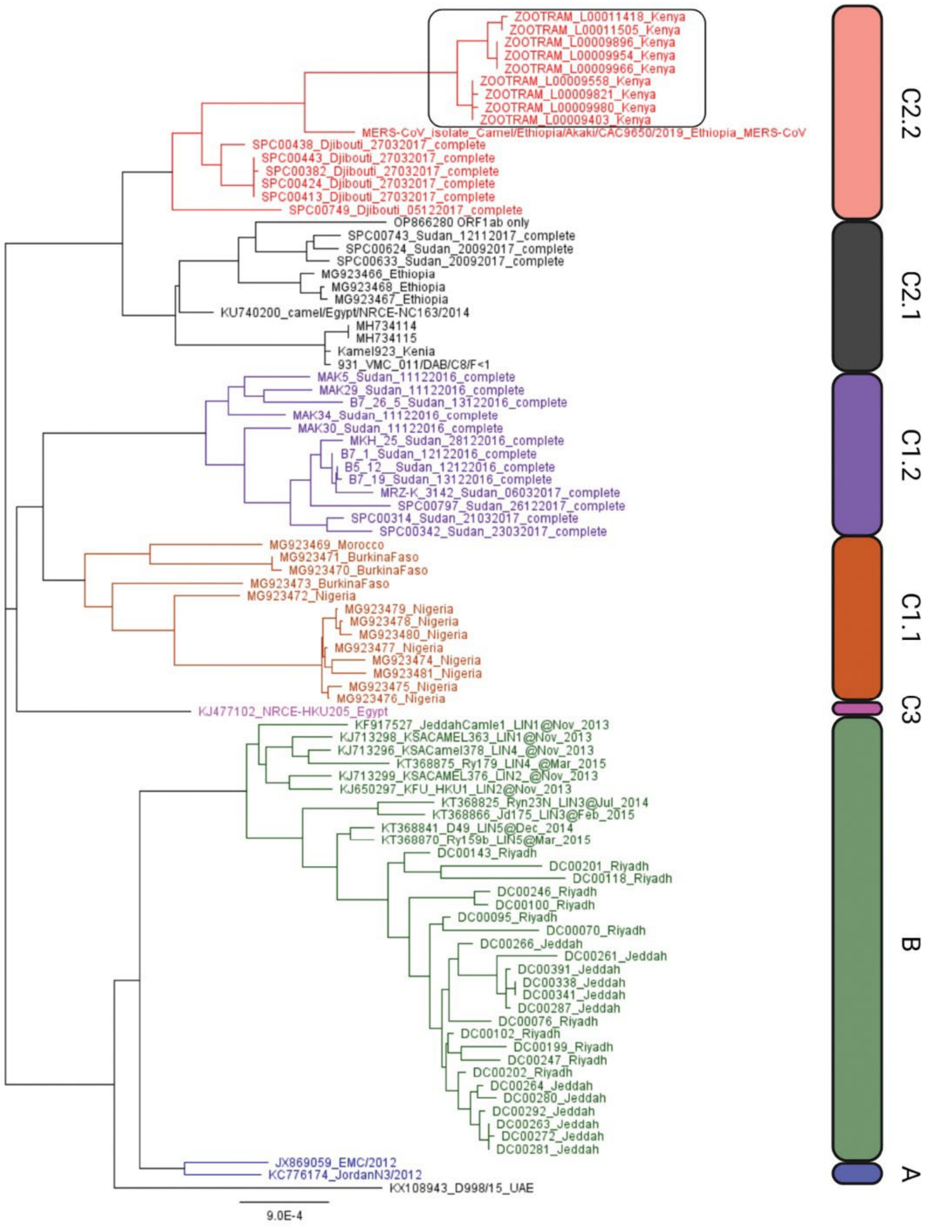

Appendix Figure 2. Phylogeny of newly identified MERS-CoV clade C2.2 strains in Kenya 2022–2023. Phylogenetic tree based on ORF1ab of MERS-CoV clade A (prototypic EMC/2012, blue), clade B (previous and contemporary MERS-CoV on the Arabian Peninsula, green) and clade C (Africa-specific MERS-CoV strains, orange (C1.1), purple (C1.2), black (C2.1), red (C2.2), magenta (C3)) reported previously (*13*,*14*). Newly identified MERS-CoV partial genomes were sequenced by NGS as described in the Material & Methods section. Sequence assembly was done by mapping the reads to the reference MERS-CoV strain (EMC/2012) using Geneious version 9.1.8 and phylogenetic analyses using Multiple Alignment using Fast Fourier Transform (MAFFT) for sequence alignment and a Tamura-Nei, neighbor-joining model for phylogenetic inference. The image was labeled using BioRender.com.

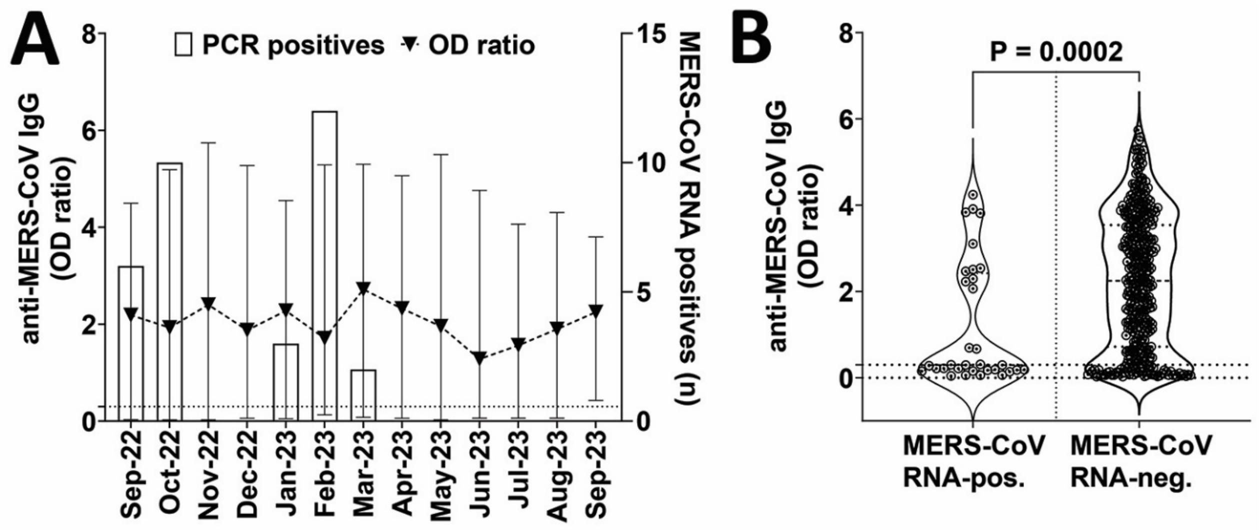

Appendix Figure 3. Overview of anti-MERS-CoV IgG (sera) and MERS-CoV upE PCR positivity (swabs) in randomized camel samples. (A) Anti-MERS-CoV S1 IgG ELISA of randomized dromedary sera (n = 369) according to the sampling month compared with MERS-CoV RNA positivity. ELISA cutoff was set to OD ratio = 0.3 according to previous studies (*4*). (B) Comparison of anti-MERS-CoV IgG levels (OD ratio) in MERS-CoV RNA negative- and positive-tested camels by MERS-CoV upE RT-qPCR (*2*). MERS-CoV RNA positivity is associated with low anti-MERS-CoV IgG levels. Statistical tests were done using GraphPad Prism (Mann Whitney test, p = 0.0002).

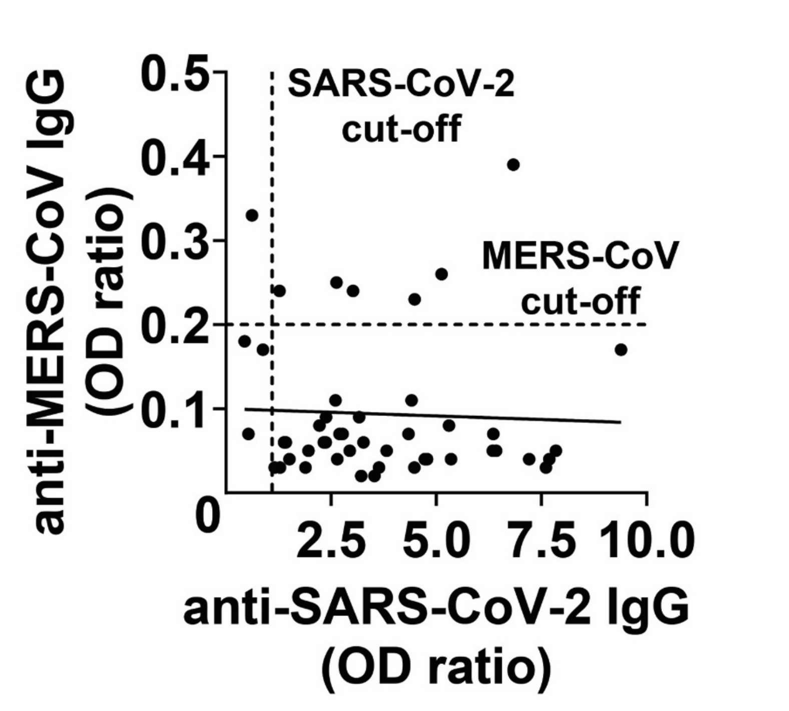

Appendix Figure 4. Antibody cross-reactivity profiling and correlation analysis of SARS-CoV-2 and MERS-CoV ELISA OD ratios in abattoir workers. The scatter plot illustrates the relationship between the SARS-CoV-2 S1 IgG ELISA OD ratio (x-axis) and the MERS-CoV S1 IgG ELISA OD ratio (y-axis) for serum samples from abattoir workers (n = 48). Dashed lines represent the cutoff values for SARS-CoV-2 ELISA (OD ratio = 1.1, vertical line) and MERS-CoV ELISA (OD ratio = 0.2, horizontal line), respectively. Data points are plotted to visually assess the correlation between the two ELISAs. The Spearman correlation coefficient ($r = -0.1449$, $p = 0.3257$) suggests a negligible linear relationship, indicating that the antibody responses to SARS-CoV-2 and MERS-CoV are largely independent ruling out a general cross-reactivity.

References

1. Corman VM, Eckerle I, Bleicker T, Zaki A, Landt O, Eschbach-Bludau M, et al. Detection of a novel human coronavirus by real-time reverse-transcription polymerase chain reaction. Euro Surveill. 2012;17:20285–91. PubMed https://doi.org/10.2807/ese.17.39.20285-en

2. Corman VM, Müller MA, Costabel U, Timm J, Binger T, Meyer B, et al. Assays for laboratory confirmation of novel human coronavirus (hCoV-EMC) infections. Euro Surveill. 2012;17:20334–43. PubMed https://doi.org/10.2807/ese.17.49.20334-en

3. Corman VM, Jores J, Meyer B, Younan M, Liljander A, Said MY, et al. Antibodies against MERS coronavirus in dromedary camels, Kenya, 1992–2013. Emerg Infect Dis. 2014;20:1319–22. PubMed https://doi.org/10.3201/eid2008.140596

4. Müller MA, Corman VM, Jores J, Meyer B, Younan M, Liljander A, et al. MERS coronavirus neutralizing antibodies in camels, Eastern Africa, 1983–1997. Emerg Infect Dis. 2014;20:2093–5. PubMed https://doi.org/10.3201/eid2012.141026

5. Drosten C, Meyer B, Müller MA, Corman VM, Al-Masri M, Hossain R, et al. Transmission of MERS-coronavirus in household contacts. N Engl J Med. 2014;371:828–35. PubMed https://doi.org/10.1056/NEJMoa1405858

6. Müller MA, Meyer B, Corman VM, Al-Masri M, Turkestani A, Ritz D, et al. Presence of Middle East respiratory syndrome coronavirus antibodies in Saudi Arabia: a nationwide, cross-sectional, serological study. Lancet Infect Dis. 2015;15:559–64. PubMed https://doi.org/10.1016/S1473-3099(15)70090-3

7. Ko JH, Müller MA, Seok H, Park GE, Lee JY, Cho SY, et al. Suggested new breakpoints of anti-MERS-CoV antibody ELISA titers: performance analysis of serologic tests. Eur J Clin Microbiol Infect Dis. 2017;36:2179–86. PubMed https://doi.org/10.1007/s10096-017-3043-3

8. Broer R, Boson B, Spaan W, Cosset FL, Corver J. Important role for the transmembrane domain of severe acute respiratory syndrome coronavirus spike protein during entry. J Virol. 2006;80:1302–10. PubMed https://doi.org/10.1128/JVI.80.3.1302-1310.2006

9. Zettl F, Meister TL, Vollmer T, Fischer B, Steinmann J, Krawczyk A, et al. Rapid quantification of SARS-CoV-2-neutralizing antibodies using propagation-defective Vesicular Stomatitis Virus pseudotypes. Vaccines (Basel). 2020;8:386–99. PubMed https://doi.org/10.3390/vaccines8030386

10. Buchholz U, Müller MA, Nitsche A, Sanewski A, Wevering N, Bauer-Balci T, et al. Contact investigation of a case of human novel coronavirus infection treated in a German hospital, October–November 2012. Euro Surveill. 2013;18:20406–13. PubMed https://doi.org/10.2807/ese.18.08.20406-en

11. Muth D, Meyer B, Niemeyer D, Schroeder S, Osterrieder N, Müller MA, et al. Transgene expression in the genome of Middle East respiratory syndrome coronavirus based on a novel reverse genetics system utilizing Red-mediated recombination cloning. J Gen Virol. 2017;98:2461–9. PubMed https://doi.org/10.1099/jgv.0.000919

12. Widjaja I, Wang C, van Haperen R, Gutiérrez-Álvarez J, van Dieren B, Okba NMA, et al. Towards a solution to MERS: protective human monoclonal antibodies targeting different domains and functions of the MERS-coronavirus spike glycoprotein. Emerg Microbes Infect. 2019;8:516–30. PubMed https://doi.org/10.1080/22221751.2019.1597644

13. El-Kafrawy SA, Corman VM, Tolah AM, Al Masaudi SB, Hassan AM, Müller MA, et al. Enzootic patterns of Middle East respiratory syndrome coronavirus in imported African and local Arabian dromedary camels: a prospective genomic study. Lancet Planet Health. 2019;3:e521–8. PubMed https://doi.org/10.1016/S2542-5196(19)30243-8

14. Zhou Z, Hui KPY, So RTY, Lv H, Perera RAPM, Chu DKW, et al. Phenotypic and genetic characterization of MERS coronaviruses from Africa to understand their zoonotic potential. Proc Natl Acad Sci U S A. 2021;118:e2103984118–26. PubMed https://doi.org/10.1073/pnas.2103984118
